# Study on the biological mechanism of urolithin a on nasopharyngeal carcinoma *in vitro*

**DOI:** 10.1080/13880209.2022.2106251

**Published:** 2022-08-11

**Authors:** Yang Yang, Zhen-Zhen Ren, Wu-Jun Wei, Zhi-Long He, You-Lin Deng, Zhuan Wang, Yu-Chun Fan, Jie Zhou, Li-He Jiang

**Affiliations:** a School of Pharmacy, Guilin Medical University, Guilin, PR China; b School of Basic Medical Sciences, Youjiang Medical University for Nationalities, Baise, PR China; c Department of Laboratory Medicine, Affiliated Hospital of Youjiang Medical University for Nationalities, Baise, PR China; d College of Light Industry and Food Engineering, Guangxi University, Nanning, PR China; e Medical College, Guangxi University, Nanning, PR China; f Key Laboratory of Tumor Immunology and Pathology (Army Medical University), Ministry of Education, Chongqing, PR China

**Keywords:** RNA-sequencing, ECM receptor interaction pathway, mitochondrial membrane potential, reactive oxygen species, apoptosis

## Abstract

**Context:**

Urolithin A (UroA) can inhibit the growth of many human cancer cells, but it has not be reported if UroA inhibits nasopharyngeal carcinoma (NPC) cells.

**Objective:**

To explore the inhibitory effect of UroA on NPC and potential mechanism *in vitro*.

**Materials and methods:**

RNA-sequencing-based mechanistic prediction was conducted by comparing KEGG enrichment of 40 μM UroA-treated for 24 h with untreated CNE2 cells. The untreated cells were selected as control. After NPC cells were treated with 20–60 μM UroA, proliferation, migration and invasion of were measured by colony formation, wound healing and transwell experiments. Apoptosis, mitochondrial membrane potential (MMP), reactive oxygen species (ROS) were measured by flow cytometry, Hoechst 33342, Rhodamine 123, JC-1 staining and ROS assay methods, respectively. Gene and protein expression were measured by RT-qPCR and Western blotting assay.

**Results:**

RNA-sequencing and KEGG enrichment revealed UroA mainly altered the ECM receptor interaction pathway. UroA inhibited cells proliferation, epithelial–mesenchymal-transition pathway, migration and invasion with IC_50_ values of 34.72 μM and 44.91 μM, induced apoptosis, MMP depolarization and increase ROS content at a concentration of 40 μM. UroA up-regulated E-cadherin, Bax/Bcl-2, c-caspase-3 and PARP proteins, while inhibiting COL4A1, MMP2, MMP9, N-cadherin, Vimentin and Snail proteins at 20–60 μM. Moreover, co-treatment of UroA (40 μM) and NAC (5 mM) could reverse the effect of UroA on apoptosis-related proteins.

**Discussion and conclusions:**

RNA-sequencing technology based on bioinformatic analyses may be applicable for studiying the mechanism of drugs for tumour treatment.

## Introduction

Nasopharyngeal carcinoma (NPC) is a common malignant tumour of the head and neck, which is more common in Southeast Asia and Southern China. Because the anatomical location of the nasopharynx is hidden and the first symptoms lack specificity, most patients have entered the middle and late stages at the first diagnosis (Lee et al. 2019). At present, the main treatment method for nasopharyngeal carcinoma is the combination of radiotherapy and chemotherapy (Sun et al. 2016). However, adverse reactions such as radiation angular stomatitis and intestinal flora disturbance are often caused, which seriously affects the quality of a patient’s daily life (Sun et al. 2016; Li et al. 2008). It is urgent and essential to investigate and find more effective therapeutic drugs to battle this lethal carcinoma.

**Figure 1.**
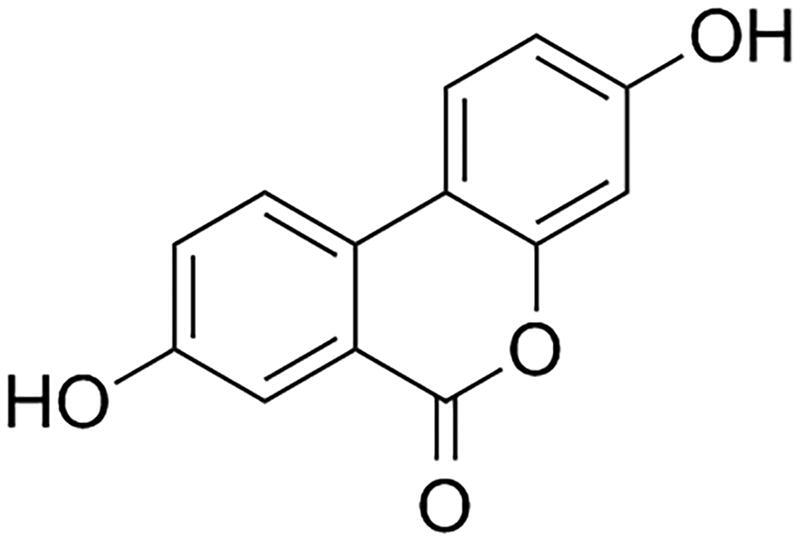
Chemical structure of UroA.

Ellagic acid mainly exists in various medicinal and edible plants in the form of ellagenic tannins (Smeriglio et al. 2017). During digestion, ellagic tannins are converted to ellagic acid, which is further metabolized by the gut microbiota into various dibenzo [*b,d*] pyrane-6-one derivatives known as urolitin (Espín et al. 2013). According to the number of hydroxyl groups and different metabolic pathways, urolithin can be divided into urolithin A (UroA), urolithin B, urolithin C, urolithin D, and methylated urolithin (González-Sarrías et al. 2010). As UroA (the chemical structure is shown in Figure 1) an intestinal metabolite, UroA has antioxidant (Mertens Talcott et al. 2006), muscle remodelling (González-Sarrías et al. 2010), anti-inflammatory (Piwowarski et al. 2015), and antitumor effects (Cheng et al. 2021).

Studies have found that UroA mediates its antitumor activity by down-regulating Wnt and IGF-1 signalling in colon cancer and prostate cancer cells (Sharma et al. 2010; Vicinanza et al. 2013), and inhibits the PI3K/AKT/mTOR pathway and has strong anti-proliferation and pro-apoptotic effects on pancreatic cancer *in vitro* and *in vivo* (Totiger et al. 2019). Furthermore, UroA can inhibit epithelial–mesenchymal transition by P53-Mdm2-Snail pathway in lung cancer cells (Cheng et al. 2021). Another study showed that UroA inhibited the proliferation of endometrial cancer cells by mediating oestrogen receptor-α-dependent gene expression (Zhang et al. 2016).

However, the exact mechanisms involved in UroA on NPC are not yet fully elucidated. Therefore, in this study, our purpose is to explore the inhibitory effect UroA on NPC and to reveal its potential mechanism *in vitro*.

## Materials and methods

### Chemicals and reagents

Urolitin A (purity ≥ 99%) was purchased from APEXBIO (Houston, TX, USA), and the storage solution was 200 mM dimethyl sulphoxide (DMSO) (Sigma-Aldrich, St. Louis, MO) at −20 °C. Cell Count Kit-8 (CCK-8) was purchased from MedChemExpress (NJ, USA); anti-E-cadherin (20874-1-AP), anti-FGFBP1 (25006-1-AP), anti-Bcl-2 (12789-1-AP), anti-MEST (11118-1-AP), anti-Vimentin (10366-1-AP), anti-N-cadherin (22018-1-AP), anti-caspase-3/p17/p19 (19677-1-AP), anti-Snail (13099-1-AP), anti-Bax (50599-2-Ig), anti-β-actin (66009-1-Ig) antibodies and secondary antibodies from Proteintech Group (Rosemount, IL); anti-PARP (#BF0719), anti-MMP2 (#AF0577), anti-MMP9 (#AF5228) antibodies from Affinity Biosciences; anti-SLFN5 (ATA37663), anti-COL4A1 (ATA27247) antibodies was purchased from Projian Bio (Wuhan, China); anti-CEACAM7 (A8112) antibodies was purchased from ABclonal Technology Co, Ltd. (Wuhan, China). Matrigel (Corning, NY, CAS:9001). Trizol (TaKaRa, Otsu, Shiga, Japan, CAS:9108). Thermo NanoDrop 2000 ultrattrace visible spectrophotometer (Thermo Fisher, Waltham, MA). PrimerScript RT kit (TaKaRa, Otsu, Shiga, Japan, CAS: RR036A). Sankong Biotechnology Co, Ltd. (Shanghai, China). 2 × ChamQ SYBR qPCR main mixture (CAS: Q311-02). Mitochondrial membrane potential assay kit with Rh123, *N*-acetyl-l-cysteine (NAC) (S0077), and Reactive Oxygen Species Assay Kit were purchased from Beyotime (Shanghai, China); FITC-Annexin V Binding Apoptosis Assay Kit from ATT Bioquest (USA); Mitochondrial membrane potential assay kit with JC-1 (Shanghai, China).

### Cell culture

Human nasopharyngeal carcinoma cells CNE1 and CNE2 were purchased from Shanghai Institute of Biological Sciences (Shanghai, China) and cultured in 1640 supplemented with 10% FBS, 100 U/mL penicillin and 100 mg/mL streptomycin at 37 °C and 5% CO_2_ in the incubator.

### Cell viability measurement

The cell viability was measured by Cell Count Kit-8 (CCK-8). The CNE1 and CNE2 cells were inoculated on 96-well plates at 2 × 10^3^ cells and incubated with different concentrations (0, 10, 20, 40, and 60 μM) of UroA for 24, 48, and 72 h. Then, CCK-8 solution (10 μL) was added to each well and the cells were cultured for 1 h. Finally, the absorbance was measured at 450 nm. The IC_50_ value was determined by drawing a linear regression curve.

### Colony formation experiment

CNE1 and CNE2 cells were seeded for colony formation in 6-well plates at a concentration of 2 × 10^3^ cells per well. After 12 days of culture, the clones were fixed with methanol for 10 min, stained with 0.10% crystal violet solution and then clones containing more than 50 cells. The colony formation rate was observed under a microscope.

### Wound healing experiment

In the wound healing experiment, CNE1 and CNE2 cells (5 × 10^5^ cells per well) were inoculated in 6-well plates and left for 24 h. After cultured, monolayer cells were scratched with pipette tip and washed once with PBS. Serum-free media containing different concentrations of UroA (0, 20, 40, and 60 μM) were added and the healing process was monitored over the next 24 h.

### Transwell chamber experiment

Longitudinal cell migration tests were performed using transwell-Boyden chambers and 8-LM well filters inserted into 24-well plates. CNE1 and CNE2 cells (2 × 10^3^ cells per well) from the serum-free medium were inoculated into the inserted filter. Cells were treated with UroA at different concentrations (0, 20, 40, and 60 μM) using a serum-containing complete medium as inducer. After incubation at 37 °C for 24 h, the cells on the upper surface of the inserted membrane were wiped with a cotton swab, and the cells on the lower surface were fixed with ethanol, stained with crystal violet, and counted under the microscope. The difference between the chamber invasion assay and the chamber longitudinal migration assay is that a layer of matrigel is applied to the upper chamber in the invasion assay, and other steps are the same as the chamber longitudinal migration assay.

### RNA-sequencing

RNA-sequencing was performed on CNE2 cells from the control group and UroA-treated group (40 μM, 24 h) using Illumina NovaSeq sequencing system (Beijing Technologies Corporation, Beijing, China). Differentially expressed genes (DEGs) were selected according to log_2_|fold change|≥1.5 and adjusted *p* value < 0.05. The KEGG pathway enrichment analysis of DEGs was conducted using the hypergeometric distribution test.

### RT-qPCR experiment

Trizol reagent was used (TaKaRa, Otsu, Shiga, Japan, CAS:9108). RNA concentrations and absorbance at 260 and 280 nm were quantified by Thermo NanoDrop 2000 ultrattrace visible spectrophotometer (Thermo Fisher, Waltham, MA). RNA integrity was determined by ethidium bromide staining on 1% agarose gel. The mRNA was immediately reversed transcript into complementary DNA (cDNA) using a PrimerScript RT kit (TaKaRa, Otsu, Shiga, Japan, CAS: RR036A) according to the manufacturer's protocol. Real-time PCR was performed in the ABI StepOnePlusTM PCR system. The primer sequences are shown in Table 1, synthesized by Sankong Biotechnology Co, Ltd. (Shanghai, China). Forward and reverse primers 0.4 µL, 50 × ROX reference dye 2 is 0.4 µL, 2 × ChamQ SYBR qPCR main mixture (CAS: Q311-02), 6.8 µL double distillation of H_2_O and 2 µL cDNA to prepare a 20 µL PCR reaction mixture. The RT-qPCR reaction was as follows: one cycle of enzyme activation and initial denaturation at 95 °C for 10 min, then 40 cycles of 95 °C for 10 s, specific primer annealing temperature is 37 °C for 30 s. A plate reading was carried out after each cycle and were normalized with the internal gene GAPDH by 2^−ΔΔCt^ method. All RT-qPCR reactions were run in triplicate. The control group was used as the calibrator. The primer sequences used in this experiment are shown in Table 1.

**Table 1.** Primer sequences used for quantitative real-time PCR in this study.

Primers	Sequences (F/R, 50–30)
CEACAM7	GTTACCCACAATGACGCAGGA TCCACCGGATTGAAGTTGTTG
ALPG	GGCATCATCCCAGTTGAGGAG TGTCACCGTAGACACCCCC
FGFBP1	GGAAACAAGTTGCCCGGAATC AATAGAGTGGAGCTGACTAGCTT
MEST	ATCGGGTGATTGCCCTTGATT GAAAGAAGGTTGATCCTGCGG
SLFN5	ACAGACGTGTCACACTGTGTT CCCTCAAGCTCGTAAGGTCTAT
DNHD1	GGCTGCGACCATTGACACT TGCTTTCATACCGTACAGGGAT
GAPDH	TTCACCACCATGGAGAAGGC TGAAGTCGCAGGAGACAACC

### Hoechst 33342 staining

CNE1 and CNE2 cells in logarithmic growth phase were inoculated at 1 × 10^5^ cells/well on 6-well plates, and cultured for 24 h. The cells were randomly divided into control group and UroA groups with 20, 40, and 60 μM, and cultured for 24 h. Hoechst staining was performed. The culture medium was removed and washed with PBS twice. The liquid was removed, 1 mL Hoechst 33342 staining buffer was added and 5 μL of Hoechst 33342 staining solution was added for 30 min. Finally, the staining solution was removed, and then washed with PBS for 2 times, 3 min/time, and the liquid was absorbed. The state of CNE1 and CNE2 cells and their apoptotic morphology were observed under fluorescence electron microscope.

### Flow cytometric analysis

CNE1 and CNE2 cells were seeded in 6-well plates at a density of 5 × 10^5^ cells/well and then exposed to UroA (0, 20, 40, and 60 μM). After 24 h, cells were harvested, fixed, washed, and stained according to the instructions in the FITC-Annexin V Binding Apoptosis Assay Kit from ATT Bioquest (USA). The stained cells were immediately analyzed by Accuri C6 Flow Cytometer (BD, Bedford, MA).

### Mitochondrial membrane potential assay

Rhodamine 123 staining was performed to measure mitochondrial membrane potential following the manufacturer’s instructions. In short, CNE1 and CNE2 cells were plated (1 × 10^5^ cells/well) in 6-wells and after UroA treatments for 24 h, cells were incubated with Rhodamine 123 fluorescent dye for 50 min in the dark. Then, the cells were washed twice with prewarmed serum-free medium and measured by a fluorescent microscope.

To quantitatively analyze mitochondrial membrane potential, we used JC-1 staining was measured by flow cytometry. In short, CNE1 and CNE2 cells were seeded in 6-well plates at a density of 5 × 10^5^ cells/well and then exposed to UroA (0, 20, 40, and 60 μM). After 24 h, cells were harvested, fixed, washed, and stained according to the instructions in the Mitochondrial membrane potential assay kit with JC-1 (Shanghai, China). Cells were stained for 30 minutes and then analyzed by Accuri C6 Flow Cytometer (BD, Bedford, MA).

### Measurement of intracellular ROS

ROS production was measured using a ROS assay kit (Beyotime Biotechnology, Shanghai, China) according to the manufacture’s protocols. Briefly, CNE1 and CNE2 cells (1 × 10^5^ cells/well) were seeded into 6-well plates overnight and treated with various doses (0, 20, 40, and 60 μM) of UroA for 24 h, respectively. Then, the cells were incubated with 10 μM DCFH-DA for 30 min followed by washing with cold PBS twice and analysis using fluorescence microscopy.

### Western blotting

CNE1 and CNE2 cells were treated with UroA at different concentrations (0, 20, 40 and 60 μM) for 24 h, and then cultured in cold RIPA lysis buffer to obtain total cell proteins. Protein concentrations were determined using a BCA kit (Beyotime, Shanghai, China). The protein samples were separated by SDS-PAGE, transferred to nitrocellulose membranes (Merck Millipore, Burlington, MA) and then sealed with fresh 5% skim milk at room temperature for 3 h. The membranes were then incubated overnight with primary antibodies at the recommended dilution at 4 °C. After washing the membranes with Tris-buffered saline and Tween-20 (TBST), they were incubated at room temperature with HRP-conjugated secondary antibodies for 1 h. The membranes probed using enhanced chemiluminescent detection reagent (Solarbio, Beijing, China) after washing. Protein expressions were normalized against β-actin by Image J Version software (BIO-RAD, Hercules, CA).

### Statistical analysis

All data were expressed as mean ± standard deviation of three independent trials. Data differences were calculated by one-way ANOVA and *t-*test. All analysis was performed using GraphPad Prism 8.0 (GraphPad software, La Jolla, CA). *p* < 0.05 was considered statistically significant.

## Results

### UroA inhibited proliferation of NPC cells

**Figure 2.**
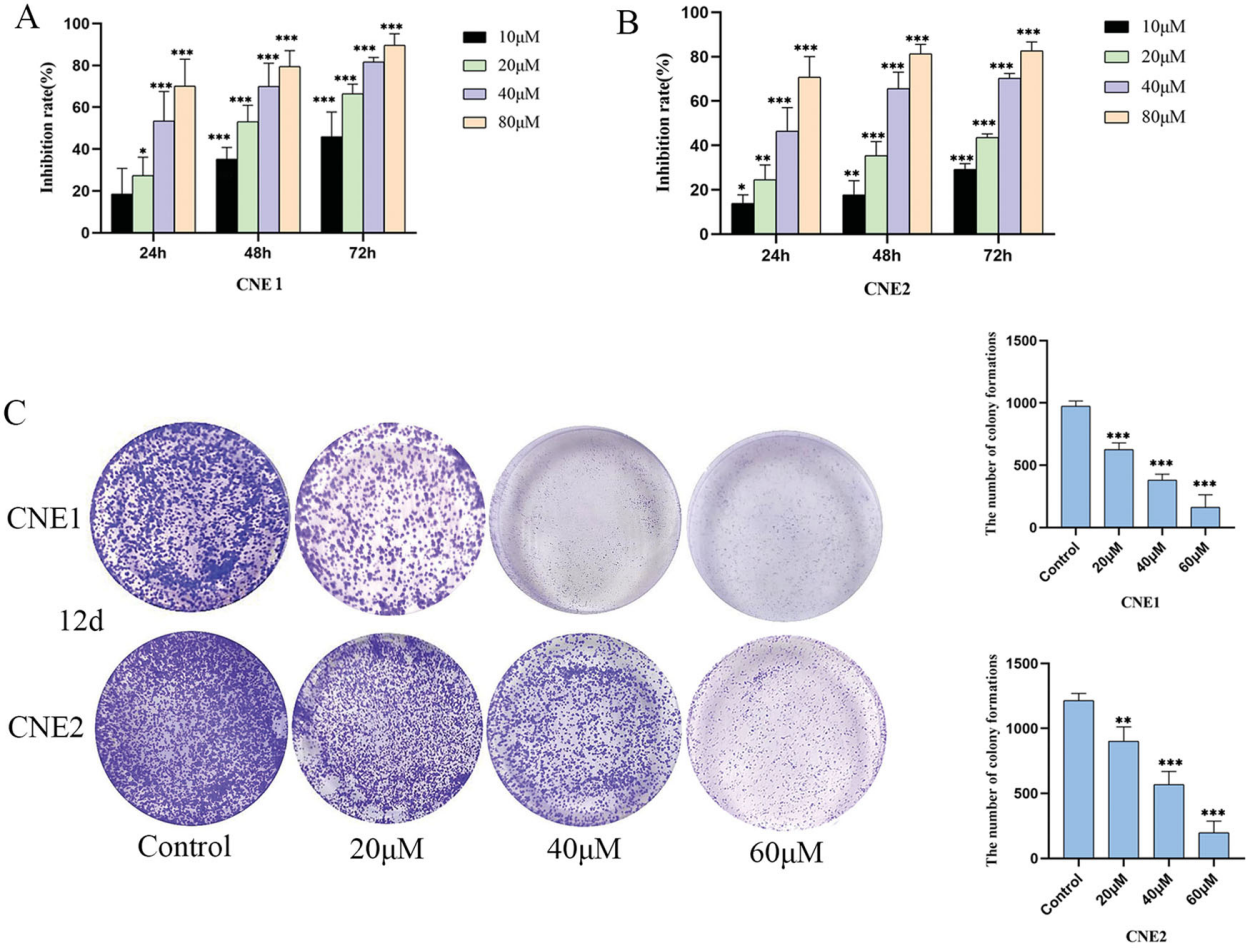
Inhibitory effect of UroA on CNE1 and CNE2 cells. (A) Inhibition rate of UroA on proliferation of CNE1 cells at indicated time points. (B) Inhibition rate of UroA on CNE2 cells at indicated time points. (C) The ability of CNE1 and CNE2 cells to form clones after UroA treatment assessed by colony formation assay. Data are shown as mean ± SD for three independent experiments, *p < 0.05, **p < 0.01 and ***p < 0.001.

A wide range of concentrations of UroA (0, 10, 20, 40 and 80 μM) were used to treat CNE1 and CNE2 cells at different time points (24, 48, and 72 h). The UroA exhibited both concentrations- and time-dependent inhibition of CNE1 and CNE2 cells, with IC_50_ values at 24 h of 34.720 μM and 44.905 μM, respectively (Figure 2(A,B)). As measured by colony formation assay, UroA significantly reduced the proliferative ability of CNE1 and CNE2 cells (Figure 2(C)).

### UroA suppressed the migration and invasion of NPC cells

**Figure 3.**
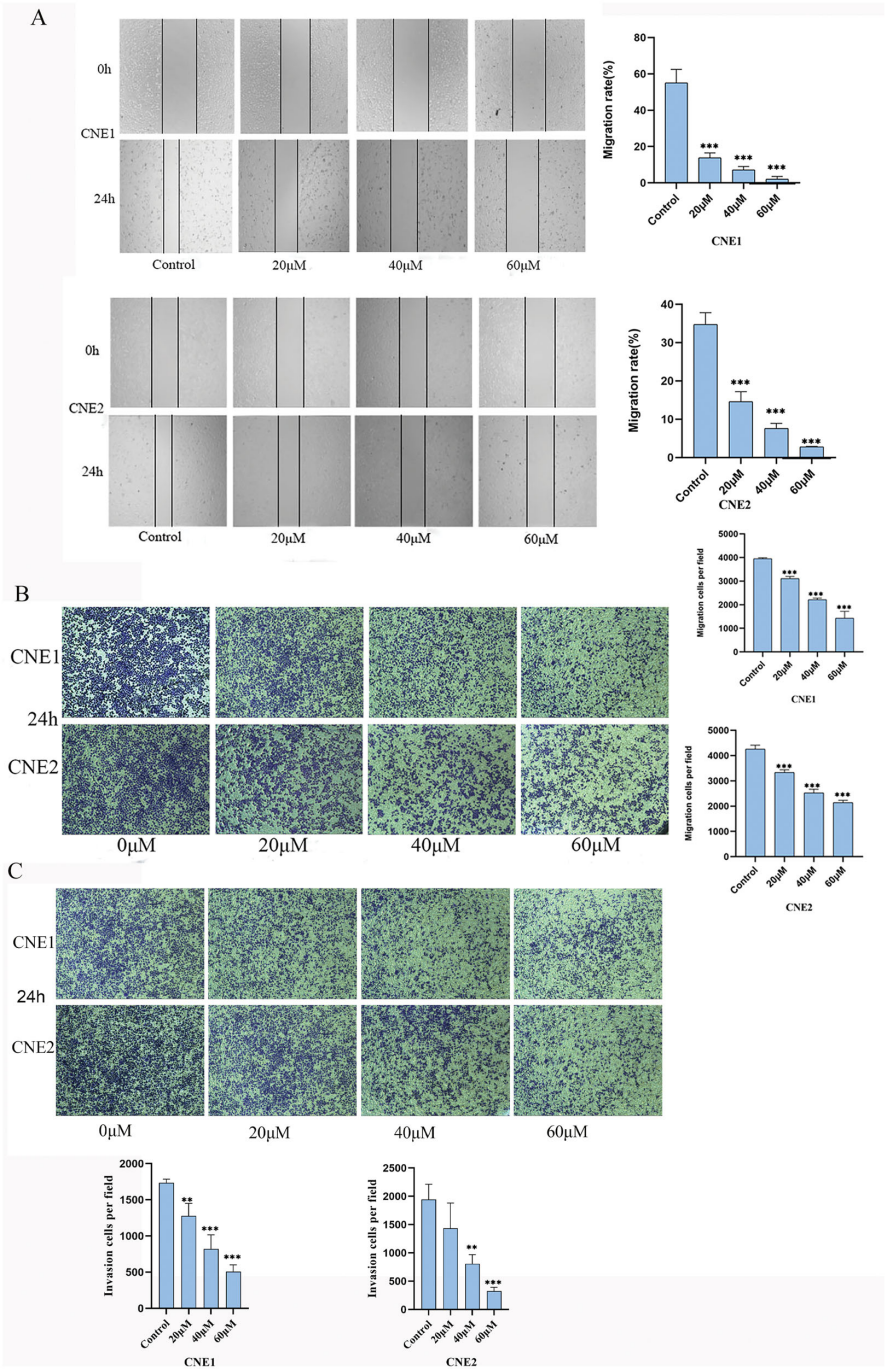
UroA suppressed cells migration and invasion of CNE1 and CNE2 cells. (A) The wound healing assay was performed to evaluate the migration ability. (B) Transwell assay was used to evaluate the effect of UroA on the migration ability. (C) Transwell assay was used to evaluate the effect of UroA on the invasion ability. Data are shown as mean ± SD for three independent experiments, *p < 0.05, **p < 0.01 and ***p < 0.001.

We performed scratch healing and transwell experiments to assess the ability of UroA on CNE1 and CNE2 cell motility. According to wound healing results, UroA significantly inhibited the migration of CNE1 and CNE2 cells in a dose-dependent manner (Figure 3(A)). Next, we examined the effect of UroA on the migration and invasion abilities of CNE1 and CNE2 cells by transwell assay. UroA significantly reduced the migration and invasion abilities of CNE1 and CNE2 cells (Figure 3(B,C)).

UroA inhibited NPC cells migration and invasion by modulating ECM receptor interaction pathway.

**Figure 4.**
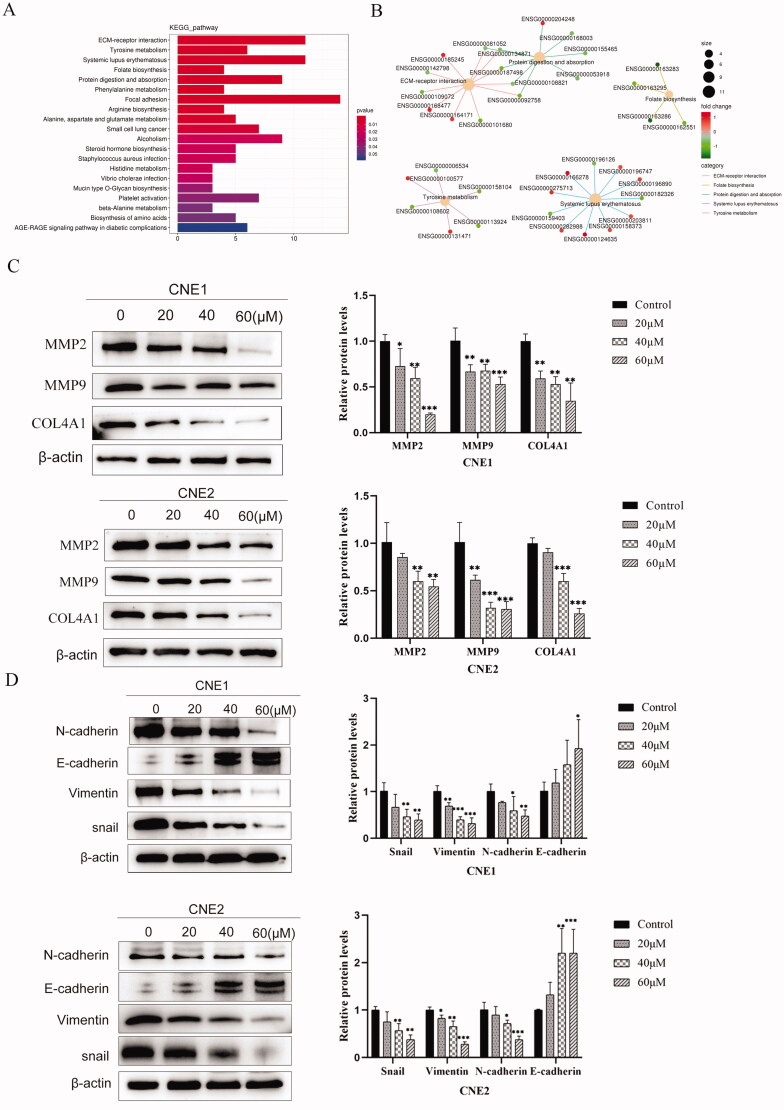
RNA sequencing and KEGG enrichment revealed ECM receptor interaction pathway mainly changed by UroA. (A) The KEGG pathway enricherment analysis of DGEs. The length of the columns reflects the number of DGEs enrichment results are more significant. Different colours represent different *P* value. (B) Co-expression network map of differentially expressed genes KEGG enrichment. (C) Western blotting analysis of COL4A1, MMP2 and MMP9 expression in CNE1 and CNE2 cells. (D) Western blotting analysis of E-cadherin, N-cadherin, Vimentin and Snail expression in CNE1 and CNE2 cells. Data are shown as mean ± SD for three independent experiments, *p < 0.05, **p < 0.01 and ***p < 0.001.

We used RNA-sequencing (RNA-seq) to investigate possible molecular mechanisms of UroA-induced inhibition of NPC cells migration and invasion. Then, used clustered Profiler package to test the statistical enrichment of DEGs in KEGG path. Pathway enrichment results indicated that UroA therapy affected several biological pathways associated with cancer, including ECM–receptor interaction, tyrosine metabolism, systemic lupus erythematosus, folate biosynthesis, protein digestion and absorption, phenylalanine metabolism, focal adhesion, etc. We found the top five pathways with the highest difference and the genes on their pathways (Figure 4(A,B)).

In order to reveal the molecular mechanism of UroA-induced NPC cell migration and invasion inhibition. Western blot was used to detect the ECM receptor interaction pathway-related proteins (Table 2). In CNE1 and CNE2 cells, compared with the blank group, UroA down-regulated the expressions of COL4A1, MMP2, and MMP9, and their expressions were lower due to the increase of UroA concentration (Figure 4(C)).

**Table 2:** Genes related to the ECM-receptor interaction pathway in the KEGG enrichment network of differentially expressed genes.

Gene ID	Gene name	Regulated
ENSG00000187498	COL4A1	Down
ENSG00000134871	COL4A2	Down
ENSG00000081052	COL4A4	Down
ENSG00000108821	COL1A1	Down
ENSG00000092758	COL9A3	Down
ENSG00000142798	HSPG2	Down
ENSG00000109072	VTN	Down
ENSG00000101680	LAMA1	Down
ENSG00000164171	ITGA2	Up
ENSG00000185245	GP1BA	Up
ENSG00000168477	TNXB	Up

The ECM has a cytoskeletal role, and changes in the arrangement of its matrix fibres can cause changes in the EMT process. In CNE1 and CNE2 cells, compared with the control group, E-cadherin was up-regulated by UroA, whereas the protein expressions of Snail, Vimentin and N-cadherin were inhibited by UroA (Figure 4(D)). Taken together, these results suggest that UroA inhibits epithelial-mesenchymal transition through the ECM.

Top differential genes regulated by UroA were verified by RT-PCR and Western blotting.

**Figure 5.**
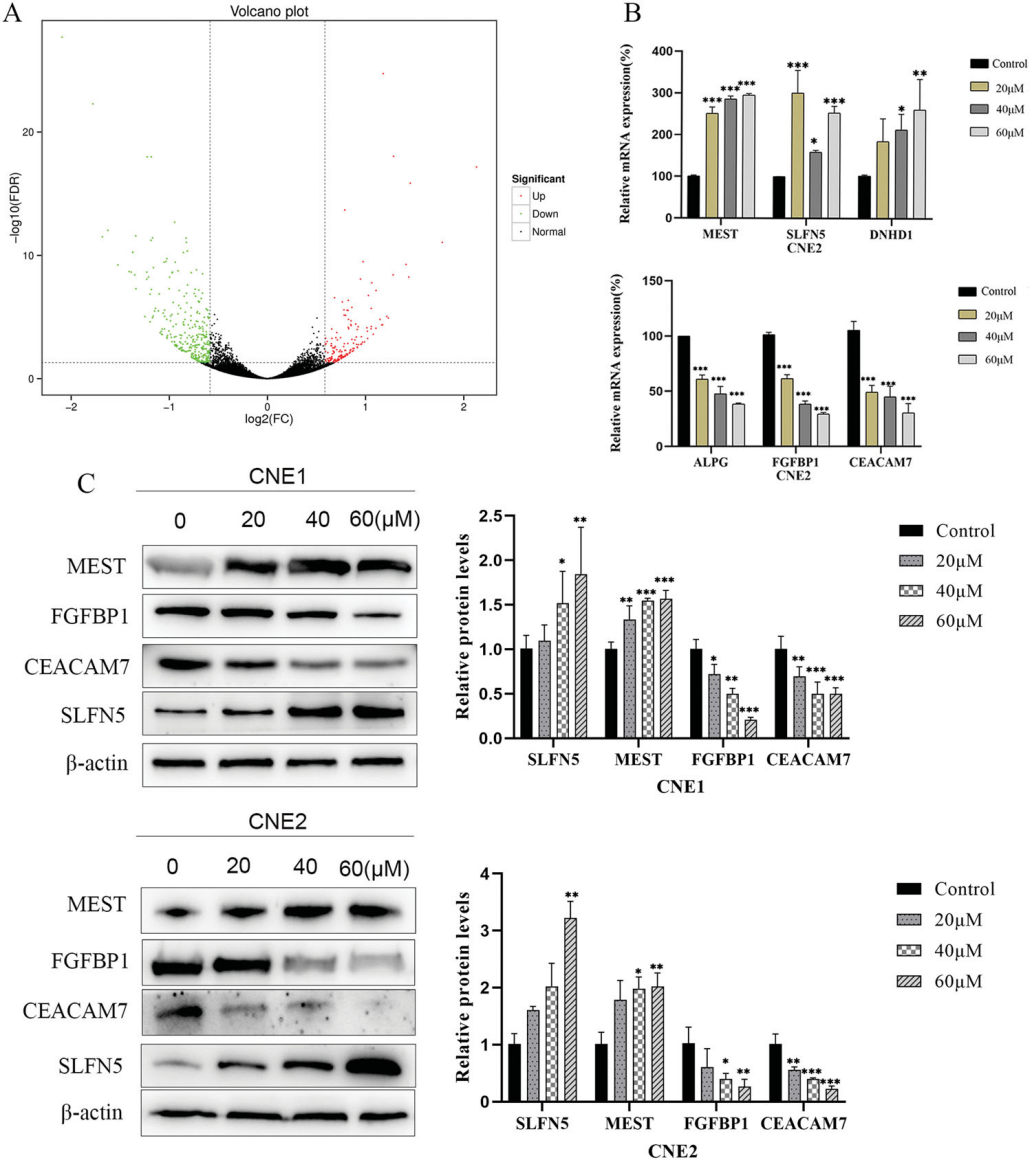
The top differential genes verification by RT-qPCR and Western blotting. (A) Volcano plot indicating up- and down-regulated mRNA transcripts in the UroA-treated group compared with the control group. (B) RT-qPCR analysis of MEST, SLFN5, DNHD1 CEACAM7, FGFBP1 and ALPG(ALPPL2), relative mRNA expression in CNE2 cells. (C) Western blotting analysis of CEACAM7, FGFBP1, MEST and SLFN5 expression in CNE1 and CNE2 cells. Data are shown as mean ± SD for three independent experiments, *p < 0.05, **p < 0.01 and ***p < 0.001.

RNA-sequencing results identified a total of 53 differentially expressed genes (DEGs, 17 upregulated and 36 downregulated) with log_2_|FC| ≥1.5 (Figure 5(A)). We listed the top 5 up-regulated genes and the top 5 down-regulated genes with the most significant differences (Table 3). RNA-sequencing showed that UroA significantly increased the mRNA expression levels of MEST, SLFN5, DNHD1 and decreased the levels of CEACAM7, FGFBP1, ALPG (ALPPL2). For further verification RNA sequencing result, RT-qPCR was used to analyzed and the results are consistent with RNA-sequencing (Figure 5(B)). Then, two high expressed genes MEST, SLFN5 and two low expressed genes CEACAM7 and FGFBP1 were verified by Western blot at protein level. After 24 h treatment with UroA, the protein expression levels of MEST and SLFN5 increased, and the protein expression levels of CEACAM7 and FGFBP1 decreased (Figure 5(C)).

**Table 3.** The genes with the most significant differential expression in the differential gene expression volcano plot.

Gene ID	Gene name	Regulated
ENSG00000007306	CEACAM7	Down
ENSG00000137440	FGFBP1	Down
ENSG00000163283	ALPP	Down
ENSG00000163286	ALPG	Down
ENSG00000177984	LCN15	Down
ENSG00000166750	SLFN5	Up
ENSG00000106484	MEST	Up
ENSG00000275395	FCGBP	Up
ENSG00000179532	DNHD1	Up
ENSG00000124635	HIST1H2BJ	Up

### UroA induced cell apoptosis via the mitochondrial apoptotic pathway

**Figure 6.**
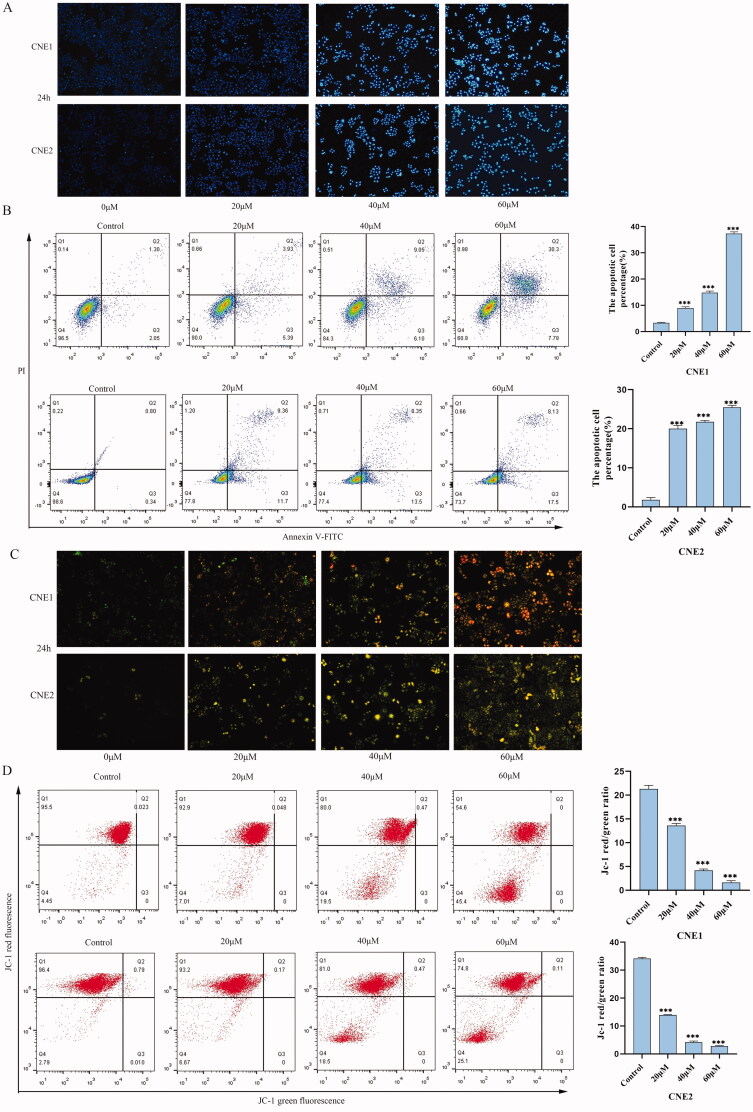
UroA induced cell apoptosis and the change of apoptosis-related proteins. (A) CNE1 and CNE2 cells were treated with or without UroA and stained by Hoechst 33342. (B) Cells were treated with UroA for 24 h and analyzed using Annexin V-FITC/PI double staining by flow cytometry. (C) The mitochondrial membrane potential alteration after the treatment with UroA were analyzed by Rh123 staining. (D) The mitochondrial membrane potential alteration after the treatment with UroA were analyzed by JC-1 staining. Data are shown as mean ± SD for three independent experiments, *p < 0.05, **p < 0.01 and ***p < 0.001.

**Figure 7.**
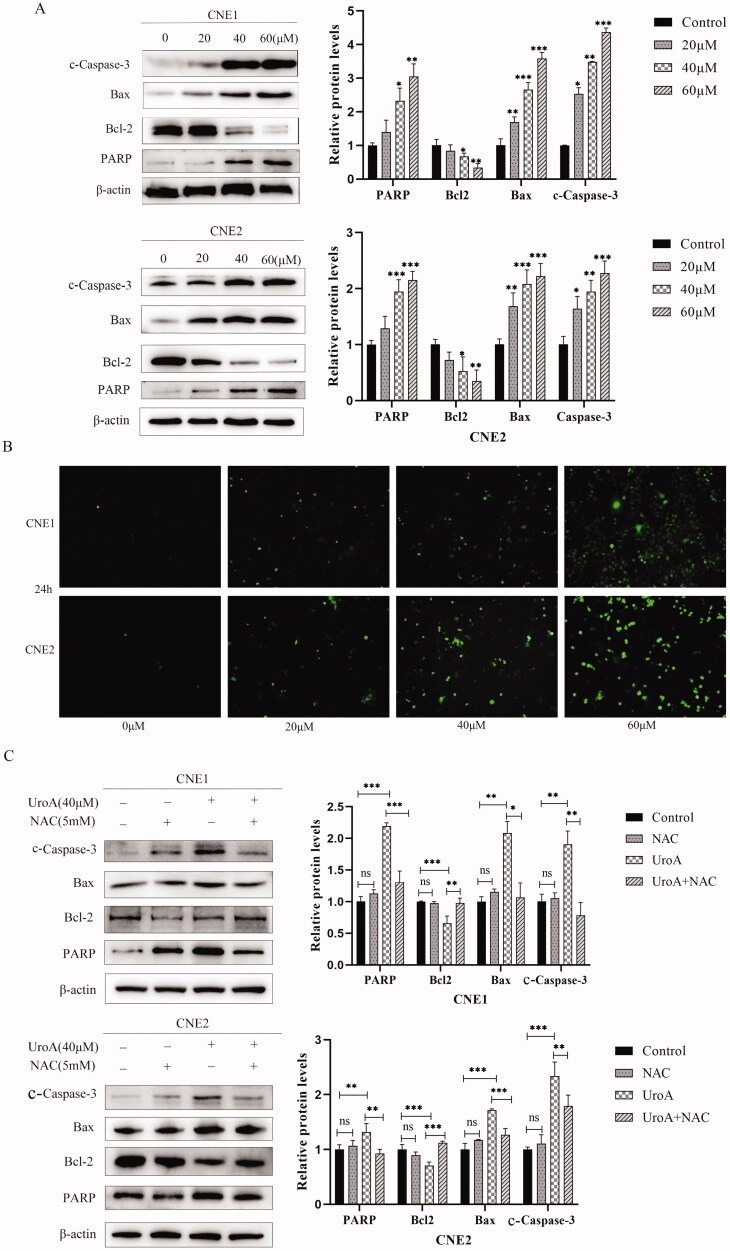
UroA induced apoptosis maybe involved in ROS-mediated mitochondrial apoptosis pathway. (A) Western blot detects the expression of apoptosis-related proteins. Data are shown as mean ± SD for three independent experiments, *p < 0.05. **p < 0.01 and ***p < 0.001. (B) Intracellular ROS levels were measured by fluorescence microscope with DCFH-DA staining. (C) Western blot was used to detect the effects of reactive oxygen species specific inhibitor (NAC) on UroA-induced apoptosis in CNE1 and CNE2 cells. Data are shown as mean ± SD for three independent experiments, *p < 0.05.**p < 0.01 and ***p < 0.001.

Hoechst33342 staining was used to study the effect of UroA on apoptosis (Figure 6(A)). CNE1 and CNE2 cells both showed bright blue fluorescence, indicating nuclear condensation and DNA fragmentation. Then, CNE1 and CNE2 cells were stained with Annexin V/PI to detect apoptosis by flow cytometry. In the UroA treatment groups, the number of apoptotic cells was significantly increased. The proportion of apoptotic cells in the CNE1 and CNE2 groups were increased from 3.35% to 38.08% and 1.14% to 25.63%, respectively (Figure 6(B)). After that we measured mitochondrial membrane potential by Rhodamine 123 staining assay, because the decrease of mitochondrial membrane potential is a hallmark of early apoptosis. The results showed that UroA-induced decrease of mitochondrial membrane potential in CNE1 and CNE2 cells (Figure 6(C)). Normal cells with higher mitochondrial membrane potential voltages to form JC-1 aggregates emit red fluorescence, while cells with reduced mitochondrial membrane potential that form JC-1 monomers will emit green fluorescence. The fluorescence ratio of red/green from JC-1 was used to assay mitochondrial membrane potential. The JC-1 assay results also showed that UroA-induced depolarization of mitochondrial membrane potential in CNE1 and CNE2 cells (Figure 6(D)). Next, we measured protein expression levels of Bax, Bcl-2, cleaved-caspase-3 (c-caspase-3) and PARP in the mitochondria associated apoptotic pathway. UroA treatment significantly increased the expressions of c-caspase-3, PARP and Bax. It also decreased Bcl-2 expression levels (Figure 7(A)).

### UroA induced mitochondrial apoptotic pathway by regulating ROS

To investigate whether UroA-induced mitochondrial depolarization is further accompanied by increased intracellular ROS levels, we used DCFH-DA to detect the intracellular ROS. We found that the content of ROS in CNE1 and CNE2 cells gradually increased with the increase of UroA concentration (Figure 7(B)). The Western blot results compared with the UroA alone treatment group, the expressions of Bax, c-caspase-3 and PARP proteins in the UroA + NAC co-treatment group were significantly decreased, and the expression of Bcl-2 was significantly increased (Figure 7(C)). It means that UroA induces mitochondrial apoptotic pathway by regulating ROS.

## Discussion

UroA is a basic metabolite produced by the intake of food and health supplements rich in ellagic acid (Totiger et al. 2019), and is a new chemical therapeutic agent with great development potential (Pei et al. 2020). Detection of cell inhibition rate and colony formation rate is a classic method to measure the effect of cell proliferation (Meng et al. 2019). In our study, we found that the CCK8 assay and the colony formation assay showed that UroA inhibited the proliferation of NPC cells. We further confirmed that UroA could inhibit the migration and invasion of NPC cells by wound healing and transwell assay. Moreover, RNA sequencing and KEGG enrichment revealed that UroA mainly altered the ECM receptor interaction pathway.

The ECM is a major component of the tumour microenvironment and can regulate the proliferation, migration and survival of tumour cells (Fang et al. 2014), the metastasis of cancer cells throughout the body may be caused by simple changes in the structure of the ECM (Park et al. 2019). The changes of ECM can affect epithelial-mesenchymal transition (EMT) in cancer cells (Ehrlich and Krummel 1996; Reig et al. 2014; Chaffer et al. 2016). Collagen IV, such as COL4A1 and COL4A2, is the most abundant component in the ECM basement membrane (Kalluri 2003; Kuo et al. 2012). Studies have shown that matrix metalloproteinases (MMPs) are involved in the physiological and pathological degradation of collagen (Van Doren 2015), MMP2 and MMP9 are two key enzymes in the process of tumour invasion and metastasis, which can help tumour cell invade through the ECM basement membrane (Niland and Eble 2020). For example, MMP-2 and MMP-9 cleave denatured and collagen IV (Löfberg et al. 1980). Our experiments showed that UroA down-regulated the protein expression of COL4A1, and decreased the protein expression of MMP2 and MMP9. Therefore, we speculate that UroA may inhibit the migration and invasion of NPC cells by regulating the EMT through the ECM–receptor interaction pathway. EMT plays an important role in cancer metastasis by promoting cell migration and invasion (Serrano Gomez et al. 2016). N-cadherin is upregulated, while E-cadherin is down-regulated during EMT in cancers (Aleskandarany et al. 2014; Miyamoto et al. 2015). In this study, we revealed that UroA restored E-cadherin expression and downregulated N-cadherin, Snail and Vimentin. We speculated that UroA has the potential to inhibit NPC cells metastasis by restraining the EMT process.

Furthermore, RNA-sequencing result showed that UroA significantly increased the mRNA the levels of MEST, SLFN5, DNHD1 and decreased the levels of CEACAM7, FGFBP1, ALPG (ALPPL2). MEST in embryonic brain cells affects neuronal migration by controlling the attachment of these cells to radial glia cells that regulate N-cadherin and N-cadherin downstream interactions (Ji et al. 2017). Knockdown of endogenous SLFN5 can induce EMT (Wan et al. 2020). DNHD1 is microtubule-based movement related protein and frequently mutated in metastatic adrenocortical cancer (Gara et al. 2018). CEACAM7 is one member of arcinoembryonic antigen-associated cell adhesion molecule belongs to the immunoglobulin family, and has cell adhesion effects (Bonsor et al. 2015). FGFBP1 can mobilize the paracrine FGF stored in the ECM and present them to their cognate receptors and upregulated in various cancers (Schmidt et al. 2018). ALPPL2 is a tumour-specific antigen (Liu et al. 2019), and is also expressed in several other tumour types, such as testicular cancer, ovarian cancer, pancreatic cancer, gastric cancer, colorectal cancer, and lung cancer (Su et al. 2020). But, the biological function of ALPPL2 in tumours is still unclear. In our study, the results showed that the mRNA expression levels of MEST, SLFN5, and DNHD1 genes were significantly increased, and the mRNA expression levels of CEACAM7, FGFBP1, ALPG (ALPPL2) genes were significantly decreased, after UroA treatment. Western blotting showed that UroA treatment can significantly increase the protein levels of MEST and SLFN5, decrease significantly the protein levels of CEACAM7 and FGFBP1. The results of these experiments are consistent with RNA-sequencing results. It is revealed that these genes are expected to become new therapeutic targets for NPC.

Mitochondria play an important role in tumorigenesis and apoptosis. Mitochondrial damage can cause cells to enter an irreversible process of apoptosis. While, mitochondrial damage is characterized by the loss of mitochondrial membrane potential (Wani et al. 2013). In our study, our experiment showed that when the concentration of UroA at 40 μM and 60 μM, the number of CNE1 and CNE2 cells apoptosis and the concentration of reactive oxygen species are increased, while the mitochondrial membrane potential was rapidly decreased.

The decreased mitochondrial membrane potential in CNE1 and CNE2 cells indicated that UroA-induced apoptosis was related with mitochondrial dysfunction. Bcl-2 and Bax cause the mitochondrial membrane permeability changes, caspase-3 is cleaved and activated subsequently, and then PARP is cleaved by caspase-3, which further regulated apoptosis (Wang et al. 2019). The exposure of 20–40 μM UroA to CNE1 and CNE2 cells resulted in the upregulating of Bax, c-caspase-3 and PARP, and down-regulating of Bcl-2. It indicated that UroA induced apoptosis follows the mitochondrial pathway.

To verify whether reactive oxygen species is involved in UroA-induced apoptosis. NAC (specific inhibitor of reactive oxygen species) was used for further studies. The results showed that the UroA + NAC co-treatment group significantly decreased the protein expression of Bax, c-caspase-3 and PARP, and the expression of Bcl-2 increased. It is speculated that UroA promotes the apoptosis of NPC cells through mitochondrial pathway by elevated reactive oxygen species content.

## Conclusion

Our research provided a new method for studying the antinasopharyngeal cancer mechanisms of UroA. As RNA-sequencing analysis predicted UroA inhibited the proliferation, migration and invasion of nasopharyngeal carcinoma cells by regulating the ECM receptor interaction pathway and inhibiting the EMT signalling pathway. UroA promotes CNE1 and CNE2 cells apoptosis, MMP depolarization and increase ROS content by ROS-mediated mitochondrial apoptosis pathway. Further study revealed that UroA can also inhibit and promote the expression of the differential proteins MEST, SLFN5, CEACAM7 and FGFBP1, respectively. We first found that UroA can inhibit the proliferation, migration, invasion and apoptosis of CNE1 and CNE2 cells. Our results provide a global view of the mechanisms of action of UroA.
